# Book Reviews

**Published:** 1807-03

**Authors:** 


### CRITICAL ANALYSIS

OF THE

### RECENT PUBLICATIONS

ON THE

### DIFFERENT BRANCHES OF PHYSIC, SURGERY, AND MEDICAL PHILOSOPHY.

---

*A practical Treatise on the Powers of Cantharides when used internally, demonstrated by Experiment and Observation, in Three Parts, including an Inquiry concerning the Nature and proper Treatment of Gleet, Leucorrhœa, and obstinate Sores.* By JOHN ROBERTON, Surgeon, Edinburgh; Extraordinary Member of the Royal Medical Society. 8vo.

A GENERAL stimulant, capable, not merely of producing temporary excitation, but of ensuring permanent support, without being followed by the debilitating consequences of some, or the narcotic effects of other powers, has long been a desideratum in the Materia Medica; a desideratum which is professed to have been supplied by the author of the work now before us.

The *Cantharis (Meloe vesicatorius Lin.)* has not been very extensively used in the practice of medicine since the days of Dr. Greenfield, but the desultory and timid manner in which it has been prescribed, is to be attributed, in the opinion of Mr. Roberton, not so much to its pernicious effects, as to the want of any precise ideas of its operation, and to a deficient acquaintance with its real efficacy.

Our author divides his work into three sections, each of which, with an absurd peculiarity, commences with a separate series of pages. The first of these sections is devoted to the consideration of Gleet; the second to Leucorrhœa; and the last to obstinate Sore; and of each, he has collected several cases for the purpose of illustrating and establishing the doctrine, that Cantharides taken internally, and prescribed judiciously, operates as an agreeable and universal stimulus to the whole vascular system, infusing not only action, but the power of maintaining action, dispelling atonia, and re-establishing health.

Mr. Roberton conceives that, in the management of Gonorrhœa and Gleets, sufficient precision has not been observed in pointing out the distinct and opposite states of both general and local excitement, which characterise these discharges from the urethra; and to this want of distinction, he, in part, attributes the contradictory accounts which have been given, respecting their mode of management. He likewise deprecates the idea of regulating the treatment of these complaints according to peculiarities which may be ascertained by microscopical inspection.

"With

"With regard to practice (says our author), it is not whether the particles discerned by the microscope, in the matter discharged, have the same shape with those of pus; it is not whether the same chemical phenomena result from trials with certain acids, nor whether the matter is infectious, that can afford much important instruction; but the information required for this purpose is, that change either in the general system, in the parts diseased, or in both, which shall render a change of treatment necessary; this appears to be the only sound basis of practical discrimination." p. 16.

To this position of our author we cordially assent. We conceive that, both in medicine and surgery, too much regard is paid to particulars, to the exclusion of generals; but cannot principles of this nature be inculcated and impressed without a dogmatism of manner, approaching to abuse? Is freedom of thought incompatible with mildness of expression? or does difference in speculative opinion, involve the necessity of intemperate and contemptuous language? In our minds, too strenuous an opposition cannot be made to this dogmatical method of conducting professional controversy; a method, which neither gives efficacy to argument, nor force to truth.

Proper Gleet, Mr. R. refers to an atonic state of the general frame, connected with deficiency of local excitation; to either direct or indirect debility; and after giving its general characters, its peculiarity, and the affections, venereal or otherwise, with which it may be combined; with cases illustrative of the advantages to be derived from the judicious employment of Cantharides taken internally; the reader is presented, in p. 132, with the following rules for its administration:

"The administration of Cantharides is to be begun in small doses, which are to be gradually increased; in the mean time we must carefully watch the changes, which proceed with such uniformity, that if our instructions be obeyed, it is our own fault if ever the patient be surprised by untoward symptoms.

"The matter of the discharge becomes gradually thick and opaque; this shews us that the inflammatory action has begun; and now we must not continue to augment the doses; but if, as sometimes happens, this appearance remains stationary, or even goes off when the dose is not increasing, then it must be increased, but very cautiously. At length an uneasy sensation is felt about the pubis; uneasiness, or even pain in the urethra, sometimes ardor urinæ, and repeated inclination to make water; the discharge has the form of laudable pus.

"At this time the doses are not to be augmented, but diminished or stopt, just as the disagreeable sensations increase in severity.

"After the use of the Cantharides is left off, if the inflammation shall abate, and the discharge become more thin, the use of the Cantharides is to be resumed, and regulated as formerly; but if the discharge gradually goes off along with the inflammatory action, the medicine is not to be repeated, for the cure will be effected without farther assistance.

"Persons

"Persons predisposed to glandular swellings, cannot use the Cantharides but with the utmost caution; and where the glands are indolent and of preternatural size, it would be very unsafe to prescribe this medicine, as inflammation and suppuration of these organs would be an almost infallible consequence.

"To those affected with pain of chest, hard, dry, or teazing cough; in short, with symptoms of tubercles in the lungs, or of incipient phthisis, the Cantharides must not be administered."

Mr. Roberton afterwards informs us, that he has never exhibited the Cantharides internally, in the form of powder, as the tincture is equally efficacious, and more manageable.

From commencing with very small doses, as in the following prescription, Mr. R. has sometimes found it necessary to give ʒvj. in the day. ℞ Tinct. Canth. ʒj. f. Aq. ſs. ℥i. M. Two teaspoonfuls four times a day.

It is observed, that where gleet or leucorrhœa has been of long standing, the patient bears much larger doses of the Cantharides than in recent cases. It is further stated, that this medicine may be continued during the period of menstruation without disadvantage.

For the purpose of subduing the unpleasant symptoms which may originate from inattention in the administration of this remedy, or from pushing it beyond due bounds, our author recommends the application of warm fomentations over the pubes, smart saline cathartics, and diluents: he doubts the peculiar accuracy of camphor, in relieving the strangury induced by Cantharides; and in another part of his work, with, perhaps, too systematic an attachment to this medicine, he questions the supposed specific tendency of it to the urinary organs; he observes, that local inflammation, as a consequence of general excitement, is sometimes obs[illegible]ved first in other parts, as is illustrated in its application to "obstinate sores."

Upon the whole, we consider this Treatise as entitled to much consideration; our attention had been called to the subject of Cantharides by a previous communication of Mr. Roberton to the Medical and Physical Journal, and we have since had opportunities of witnessing its frequent, if not invariable success, in the complaints to which our author has appropriated it. Perhaps the range of diseases is still more extensive, in which a judicious and very circumspect employment of Cantharides might prove of considerable advantage.

---

*Oratio in Theatro Collegii Regalis Medicorum Londinensis, ex Harveii Instituto, habita; Die Octob.* xviii, *An.* 1786. A CHRIST. ROB. PEMBERTON, M. D. Principi Walliæ Medico Extraordinario, Colleg. Reg. Med. Lond. et Reg. Soc. Socio. Londini, apud Nicol. 1807.

AFTER an exordium, in which the orator, according to the custom of other speakers, regrets with much modesty the task imposed posed upon him, some difficulties arise with whose name he shall begin, where there are so many who deserve notice. Linacre is preferred as the founder of the Institution, which is described in the following summary way :—Medicinæ Doctorum ordinem, qui omnibus hanc artem exercentibus intra Londinum et circumcirca septem millia passuum, auctoritate summa, præessent; et omnium etiam, qui artem professi per Angliam dispersi fuerint, studia eorum percontarentur. From Linacre to Harvey, we have only the names of Caius and Gouldstone, to each of whom the College acknowledges obligations. Harvey is introduced, described, and dismissed, in a manner worthy of himself, as far as justice can be done to so illustrious a character. Glisson follows, as the first who attempted to ascertain the cause of disease by inspection immediately after death. Most of these names, it appears, are introduced on account of the services they rendered the College. But now a just preamble introduces the illustrious Sydenham, during whose history we hear nothing of the college; we may therefore, suppose, that he never arrived at the honour of a fellowship. Probably the law, by which the president has a power to introduce a licentiate, did not in those days exist. The account of Sydenham's character seems short, but so would every account however long. It is generally well expressed, and one sentence we were particularly pleased with : Res ipsas ipse inspexit, earumque discrimina sub eodem nomine latentia mirifico quodam acumine detexit et distinxit. It is afterwards observed, that Sydenham was the founder of a new order of physicians, who taught the study of nature, instead of giving a fancied importance to trifles; we sincerely wish he had been as generally imitated as he has been justly praised.

The names of Morton, Cheyne, Friend, Arbuthnot, Garth, and Radcliffe, with an etcetera, are slightly passed over to introduce Mead, of whose medical abilities, the Orator finds it difficult to say much; but this is in some degree compensated in describing the accomplished scholar and contemporary of Newton and Halley. From Mead, we meet with no name till Heberden is introduced with the decency, respect, and gratitude he deserves. Next to him follow Turton and Gisborne, of whom, nothing more is said, than, that they were King's Physicians, and that the latter was a great stickler for the College. This introduces an apostrophe, which is so much like, "*the church is in danger*," that it is impossible not to smile whilst we read it.

"Quis vestrum ignorat, alienorum hominum consessum habitum esse, novis consiliis, nova audacia erectum, ad reformandam, ut aiunt, vel potius evertendam eam Medicam disciplinam, quæ in hac nostra domo per tria secula feliciter constituta est. Immo eo processit hæcce rerum novarum cupiditas, ut consulerent de petitione Senatui inferenda, ad inceptum suum lege sanciendum. In tali causa ubi is vestrum invenitur, *qui non ad arma currat?* —Quis non clamet "Stet fortuna Domus," clamandoque pro salute lute nostræ reipublicæ propugnet? Anne antiquam illam Majorum domum, quæ talem heroum progeniem quasi in gremio aluit, dirui tandem et collabi patiemur? Uno animo statuimus omnes, pugnandum esse pro hac nostra patria—asserenda quæcunque sunt jura—vindicanda privilegia—tenendosque mores a patribus receptos. Neque deficiunt nobis periti viri amantesque nostrum, qui causam, contra hostium insidias, advocati tuebuntur: quinimo etiam et duce gloriamur, huic nostræ civitati Præfecto, quem nullæ rerum angustiæ exanimant, nulli labores fatigant, quem in consulendo gravissimum, in agendo promptissimum esse cognovimus."

For the honour of the profession however, and the credit of the Orator, we must add the succeeding sentence, which, we need not inform our readers, relates to Jenner and his discovery.

"Quanquam certis quibusdam præscriptisque finibus hujuce Orationis præconium concludatur, non possum quin, extra terminos paululum evagando, vobis proponam rem nuperrime exploratam, quæ, si firmo nitatur fundamine, omnibus aliis inventis longissime præcellit, qualia e primordiis rerum ad hominum conditionem sublevandam unquam fuerint excogitata."

An elegant panegyric on the Inventor follows, which induces us to trust, that the College only waits the decision of the grand question to number Jenner among their Fellows, unless they still fear the *Fortuna Domus.* It is evident that every means but those worthy of such an act, and of such an establishment, must prove as futile, we will not use a harsher word, as that flourish of martial music—*Quis non ad arma currat!*

---

*An Account of a new discovered Membrane in the Human Eye.. To which are added, some Objections to the common Operation for Fistula Lacrymalis; and the Suggestion of a new Mode of Treating that Disease.* By S. Sawrey, Surgeon. Quarto. London, 1807.

The newly discovered Membrane, if we perfectly understand the author, is a lining to the inside of the transparent cornea, and attached at its extremities to the edge of the iris. If such is the case, the aqueous humour does not come in contact with the cornea lucida. The author even suspects, that another fluid may be interposed between the transparent cornea and the membrane he has discovered. On this subject, however, we shall be silent, aware, that a matter of fact can only be proved or disproved by demonstration; and those, who have leisure and diligence to pursue the inquiry, will avail themselves of all the assistance which the work itself affords.

On the subject of Fistula Lacrymalis, the author shows the defects of every operation hitherto proposed, and then points out a method, which, he conceives, may be more successful. Under the first division, we meet with a candid well digested history of the various theories concerning the nature of the disease, and the consequent mode of treatment. The uncertainty of all, and the probable success of most, induce him to conclude, that nothing more has hitherto been done than to cicatrize the ulcer, and, in most instances, destroy the duct. It seems, indeed, probable, that whenever the canal has been preserved, the patient has owed more to the progress of Nature, than to any assistance from art.

The new method consists of attempting to remove any obstruction in the duct, by passing a small probe from the nose upwards. This seems to have its advantages; for if a probe cannot be passed through the whole canal, it would be better to dislodge any obstruction at the bottom than at the top. However, the author writes with so much perspicuity and modesty, that we shall transcribe the passage.

"The advantage of this mode, if practicable (and that it is so, will appear from the following case), must appear evident to every one. The canal is of such a magnitude, in the healthy state, as to be capable of receiving a common sized probe; but in the mode commonly recommended, of introducing one at the puncta lacrymalia, the probe must be so small, and the way is so circuitous, that little can be expected from it; nor can much be hoped for from injection by Anel's syringe, at least where the obstruction is of a permanent nature.

"Of late, finding many and great objections to the common operation for this disease, I ventured to try to introduce a probe, in the way I am recommending, in the case of a lady who had a fistula lacrymalis.

"In this instance, I had not only succeeded in getting it into the nasal duct, but the introduction was attended with little or no pain, nor did it even cause any unpleasant sensation, or sneezing, which I expected it would have done. The advantage to the complaint was also great and immediate, as will be seen from a relation of facts.

"Oct. 15th, 1806. Miss —— applied to me for a weakness of sight.—The left eye was generally full of water, and vision only perfect for a few seconds, after absorbing the moisture with a handkerchief. The vessels on the ball of the eye, towards the inner canthus, were very conspicuous; the lids were glued together in the morning. She complained of a dryness of the nostril on that side, and that she could not breathe so well through it as usual. When the lacrymal sac was pressed, tears, mixed with puriform matter and mucus, regurgitated into the eye.

"About eight years ago, a tumour formed in the inner corner of the eye, which burst and discharged matter for a considerable time; since which time, the above-described symptoms have been present. After two or three trials with a curved probe, of the common size, I found I was only able to enter the nassal canal for a little way, the probe feeling as if embraced tightly by the canal.

canal. I, therefore, had a smaller probe made; and on the 22d, by gentle pressure, I felt the end of the probe suddenly pass through two different obstructed parts, and got it up at a considerable distance, and with very little pain. On withdrawing it, a good deal of mucus adhered to the probe, and the patient found something trickle in her nose and throat, which she feared was blood; but, on blowing her nose, it was found to be water: she expressed much relief, feeling the nostril much more moist, and as if something had been removed, which enabled her to breathe through it more freely.

"25th.—The weeping had become much less, but more particularly so on the day on which the probe was introduced. The eye-lids did not adhere near so much in the morning, nor were the vessels on the inside of the ball of the eye so conspicuous. I again introduced the probe with great ease, and kept it in for a minute or two. When the lacrymal sac was pressed, she observed that she felt the probe to be moved.

"26th—She felt almost quite well all day yesterday: when stooping forwards two or three times, water dropped from that nostril, which feels much more moist: she could blow the nose with greater ease: the eye looked considerably better. On pressing the sac, I could hardly perceive any regurgitation, and that only of water and a little mucus. I now introduced a probe of a rather larger size; when the bulbous part reached the angle of the eye, this became filled with water, which disappeared in a minute or two after withdrawing the instrument; water also ran down the probe whilst in the canal.

"Nov. 16th.—The probe had been introduced only twice since the 26th. The eye did not appear quite so well; I introduced the probe, and afterwards threw up a little luke-warm water, by a common syringe, fitted to a tube of the same dimensions and curve as the probe. By this means, I forced the water into the eye; some of it also escaped into the throat.

"18th.—She was much better: I repeated the operation of probing and syringing. Dec. 1st. Repeated the operation of syringing and probing four or five times since the last report. She seemed well;—no watery appearance, dryness of the nostril, nor gumming up of the eye-lids; I purposed to repeat the operations of probing and syringing for some time.

"It is to be observed, that although the good effects of the practice, in this particular case, was so decisive, yet, from the disease having advanced to suppuration, it was not the most favourable for the operation. In an incipient state, one might more reasonably expect the same, or still greater benefit; for when suppuration once takes place, the structure of the surrounding parts becomes much altered.

"I am aware, that the introduction of the probe is not altogether unattended with difficulty, and in some cases more than in others. It not only requires an exact knowledge of the parts, but also also a quick sensation of touch, and some address or knack on the part of the practitioner, which is not easily explained. However, I am confident that a little experience will overcome the difficulty, and that the practice will tend greatly to promote the cure of the disease in question."

---

*Cases and Cures of Hydrophobia, selected from the Gentleman's Magazine: containing many curious and interesting Accounts relative to that most alarming Malady.* London, 1807.

THOUGH the late, we trust unfounded, alarm, has given rise to this selection, yet the public will have reason to thank the diligence of the compiler. A mass of evidence and facts is collected, which is always useful, were it only to show the insufficiency of remedies, which, on these melancholy occasions, will always be proposed, and to stimulate us in the inquiry after others. We shall avoid any selection, suspecting that every claim to success has proved fallacious, from the remedy falling afterwards into disuse; but we recommend our medical readers to make an index of the various modes of treatment, that they may be at once referred to whenever these unhappy cases occur.

We shall transcribe one passage, though it has already appeared in two different periodical works.* The importance of ascertaining the symptoms in the dog is so great, that a knowledge of them cannot be too generally diffused. The description which follows is Mr. Menel's, whose acquaintance with every thing relative to the kennel is universally admitted.

"The first symptoms of canine madness in dogs is, I believe, a failure of appetite in a small degree; I mean, that the dog does not eat his usual food with his usual eagerness: though, if better food be offered him, he may eat it greedily. A disposition to quarrel with other dogs, comes on early in the disease. A total loss of appetite generally succeeds; though I have seen dogs eat and lap water the day before their death, which generally happens between seven and ten days after the first symptom has appeared. A mad dog will not, I believe, cry out on being struck, or shew any sign of fear on being threatened, though he will, very late in the disease, appear sensible of kind treatment.

"I have never known a mad dog shew symptoms of the disease in less time after the bite than ten days; and I have known many instances of dogs having died mad as late as eight months after the bite I think the symptoms generally appear between three and eight weeks after the bite.

"A mad dog, in the height of the disorder, has a disposition to bite all other dogs, animals, or men. When not provoked, he usually

---

* Medical and Chirurgical Memoirs, vol. i.; and Duncan's Medical Comment. vol. xix.

usually attacks only such as come in his way; but, having no fear, it is peculiarly dangerous to strike at or provoke him.

"Mad dogs appear to be capable of communicating the affection early in the disorder, and as soon as they begin to quarrel with or bite other dogs.

"The eyes of mad dogs do not look red or fierce, but dull, and have a peculiar appearance, which is easily distinguished by such as have been used to observe it, but not easy to be described.

"Mad dogs never bark, but occasionally utter a most dismal and plaintive howl, expressive of extreme distress; and which they who have once heard can never forget. So that dogs may be known to be going mad, without being seen, when only this dismal howl is heard.

"Mad dogs do not foam or froth at the mouth, but their lips and tongue appear dry and foul, or slimy.

"Though mad dogs generally refuse both food and drink in the latter stage of the disorder, yet they *never show any abhorrence or dread of water; will pass through it without difficulty, and lap it eagerly to the last.* But it is remarkable, that though they lap water for a long time, and eagerly, and do not seem to experience any uneasiness from it, yet they do not appear to swallow a single drop of it; for, however long they may continue lapping it, no diminution of quantity can be perceived.

"I am persuaded, that this disorder never originates from hot weather, putrid provisions, or from any other cause but the bite: for, however dogs may have been confined, however fed, or whatever may have been the heat of the season, I never knew the disorder commence without being able to trace it to that cause; and it was never introduced into the kennel but by the bite of a mad dog.

"The hairs of a mad dog do not stand erect more than those of other dogs. I do not know that there is any thing remarkable in the manner of a mad dog's carrying his head or his tail. I do not believe that dogs are more afraid of a mad dog than they are of any other dog that seems disposed to attack them.

"There are two kinds of madness, both of which I have known to originate from the bite of the same dog. Among huntsmen, one is known by the name of raging, the other by that of dumb madness. In dumb madness, the nether jaw drops, and is fixed, the tongue hangs out of the mouth, and slaver drops from it. In raging madness, the mouth is shut, except when the dog snaps or howls, and moisture drops from it."

---

*A Letter to the Editor of the British Critic; occasioned by some Remarks, in that Review, on a Book entitled Cases of Pulmonary Consumption, &c. treated with Uva Ursi.* By the Author of the above-mentioned Book. Oxford, 1806.

How often do we hear scholars quote

— Ingenuas didicisse fideliter artes

Emollit mores, &c.

The task of a Critic was once considered as a most sacred duty. Certain laws were established, and when these were found too severe for general application, a kind of courtesy was introduced, by which, a master of the art was expected to remind his author, instead of accusing him; to instruct, instead of exposing him. In the progress of knowledge, however, from the extension of copies by the art of printing, the number of authors became so great, that a Review of them, without the minuteness of close criticism, became necessary. As these works soon became the property of booksellers, who employed the most industrious labourers, it is not to be wondered, if the employment was very much confined to the poorer class of scholars. As these are pretty universally disposed to overvalue learning, and, perhaps, to feel a portion of dissatisfaction, at seeing offices of emolument, particularly in the church, filled by such as they conceived their inferiors in literature, it is not to be wondered if many of their remarks were indirectly pointed against established forms, and even constituted authorities. This was particularly charged on the Monthly Review, which, in other respects, was so well conducted as to secure the public patronage for full half a century.

At length, the importance attached to the writings of Philosophers, and the influence they were supposed to maintain with the public, seemed to call aloud for some remedy which might counteract a growing disease. Though Priestly was as weak in controversy as he was strong in philosophy, yet it was thought necessary to do more than to show the flimziness of his polemics. He and Price were both sectaries, and the Monthly Reviewers were their friends. It is true, the former had been roughly handled by his supposed associates, but not so roughly as his adversaries thought it necessary; and the Christian meekness of the latter, seemed to set all abuse at defiance.

Under these circumstances, to fight the enemy with his own weapons was absolutely necessary, and two men were selected, who were certainly competent, as far as scholastic knowledge extends. The new Review, as Dr. Bourne observes, "was, perhaps, in an especial manner circulated among the members of the University." What, let us ask, was the cause of this particular circulation? Was it because the writers showed themselves qualified for a *7th class*? Was it on account of their brilliancy of wit, strength of erudition, or close adherence to truth, which last last should be the ultimate object of all academic researches? Let Dr. Bourne put his hand upon his heart, and ask his brethren on the banks of the Isis, whether they ever gave themselves the trouble to enquire, whether any of the allegations against Priestly were true? Whether they did not greedily swallow all the ungentlemanly ribaldry against honest men, or men who should be supposed honest, according to the courtesy of our constitution. Are then medical truths more important than religious truths? or are medical men more important than those who consecrate themselves to religion. Dr. Bourne, when he reflects on the character of the Review, in which he conceives himself ill-treated, should rather feel contented that he has escaped so well, unless he conceives some respect due to him as a member of the University. But he should reflect, that neither of the two acknowledged Editors of the British Critic is a medical man; and as this kind of labour is generally done as cheap as it can be bargained for, probably, a drudge has been selected who has little respect for the Castalian fountains.

We perfectly agree with Dr. Bourne, that a Reviewer "is bound, both by the ties of honour and of morality, to state the facts and sentiments which he produces as from the Author, with truth and fairness; to be accurate in his quotations; and to give no other sense to a quotation, than the sense that it obviously bears, when taken in conjunction with the context. I think moreover, adds Dr. B. that he is not at liberty to introduce, *as his own*, those observations, for which he is indebted to the book under review."

But has the British Critic scrupulously attended to these precepts in reviewing other works? We must remind our author, once more, that his business should have been to explain to the critics, that their medical reviewer is not the man they ought to employ; since he dares to depreciate the works of one, whose graduations, fellowships, and profesosrships, should entitle him to respect. We are ready to admit, that Dr. Bourne has a much more solid cause of complaint in the injustice with which he has been treated. But for justice he should appeal to the Public; for academic privileges, to the British Critic.

